# Mortality in vegetarians and comparable nonvegetarians in the United Kingdom^1^,^2^,^3^

**DOI:** 10.3945/ajcn.115.119461

**Published:** 2015-12-09

**Authors:** Paul N Appleby, Francesca L Crowe, Kathryn E Bradbury, Ruth C Travis, Timothy J Key

**Affiliations:** Cancer Epidemiology Unit, Nuffield Department of Population Health, University of Oxford, Oxford, United Kingdom

**Keywords:** diet, nonvegetarian, vegetarian, vegan, mortality

## Abstract

**Background:** Vegetarians and others who do not eat meat have been observed to have lower incidence rates than meat eaters of some chronic diseases, but it is unclear whether this translates into lower mortality.

**Objective:** The purpose of this study was to describe mortality in vegetarians and comparable nonvegetarians in a large United Kingdom cohort.

**Design:** The study involved a pooled analysis of data from 2 prospective studies that included 60,310 persons living in the United Kingdom, comprising 18,431 regular meat eaters (who ate meat ≥5 times/wk on average), 13,039 low (less-frequent) meat eaters, 8516 fish eaters (who ate fish but not meat), and 20,324 vegetarians (including 2228 vegans who did not eat any animal foods). Mortality by diet group for each of 18 common causes of death was estimated with the use of Cox proportional hazards models.

**Results:** There were 5294 deaths before age 90 in >1 million y of follow-up. There was no significant difference in overall (all-cause) mortality between the diet groups: HRs in low meat eaters, fish eaters, and vegetarians compared with regular meat eaters were 0.93 (95% CI: 0.86, 1.00), 0.96 (95% CI: 0.86, 1.06), and 1.02 (95% CI: 0.94, 1.10), respectively; *P*-heterogeneity of risks = 0.082. There were significant differences in risk compared with regular meat eaters for deaths from circulatory disease [higher in fish eaters (HR: 1.22; 95% CI: 1.02, 1.46)]; malignant cancer [lower in fish eaters (HR: 0.82; 95% CI: 0.70, 0.97)], including pancreatic cancer [lower in low meat eaters and vegetarians (HR: 0.55; 95% CI: 0.36, 0.86 and HR: 0.48; 95% CI: 0.28, 0.82, respectively)] and cancers of the lymphatic/hematopoietic tissue [lower in vegetarians (HR: 0.50; 95% CI: 0.32, 0.79)]; respiratory disease [lower in low meat eaters (HR: 0.70; 95% CI: 0.53, 0.92)]; and all other causes [lower in low meat eaters (HR: 0.74; 95% CI: 0.56, 0.99)]. Further adjustment for body mass index left these associations largely unchanged.

**Conclusions:** United Kingdom–based vegetarians and comparable nonvegetarians have similar all-cause mortality. Differences found for specific causes of death merit further investigation.

See corresponding editorial on page 3.

## INTRODUCTION

Vegetarians are defined as people who do not eat any meat, poultry, or fish. They may be subclassified as lacto-ovo-vegetarians, who eat dairy products and/or eggs, and vegans, who do not eat any animal products. Others choose to limit their consumption of meat and fish, perhaps eating fish but not meat (often described as pesco-vegetarians) or eating meat infrequently (sometimes described as semivegetarians). Vegetarianism is uncommon in most countries, with <10% of the population following a vegetarian diet (1). For example, vegetarians and vegans have been estimated to constitute 5% and 2%, respectively, of the US population (2). In India, the proportion of the population consuming a vegetarian diet is much higher at almost 30% (3). These figures indicate that vegetarians and others who eat little or no meat represent a sizeable minority of the global population, and with calls for a reduction in the average worldwide consumption of animal products (4), their long-term health is a matter of considerable interest.

Previous studies of mortality in vegetarians have not consistently shown a difference in overall mortality between vegetarians and comparable nonvegetarians (by which we mean persons from a similar background to vegetarians, including ethnicity and socioeconomic status, who eat meat and/or fish). For example, in a pooled analysis of 5 prospective studies, the death rate ratio in vegetarians compared with nonvegetarians, based on a total of 8330 deaths, was 0.95 (95% CI: 0.82, 1.11), albeit with significant heterogeneity of risks between studies (5). Further analyses of mortality data from the European Prospective Investigation into Cancer and Nutrition (EPIC)^4^–Oxford study (6) and Adventist Health Study 2 (AHS-2) (7), based on 1513 and 2570 deaths, respectively, produced inconsistent results (8, 9). In the EPIC-Oxford study, there was no difference in all-cause mortality between vegetarians and nonvegetarians (HR: 1.05; 95% CI: 0.93, 1.19), although the overall death rate was only one-half that of the United Kingdom population as a whole (8). In contrast, all-cause mortality in the AHS-2 was 12% (95% CI: 3, 20) lower in all vegetarians combined than in nonvegetarians (9). However, heterogeneity of risks between studies (5) and small numbers of deaths from specific causes have limited the ability of researchers to study relative mortality for many common causes of death.

To provide more information on mortality in vegetarians and vegans, persons who eat fish but not meat, and infrequent meat eaters, we report here HRs for each of 18 common causes of death, including all causes combined. The analysis used pooled mortality data from 2 prospective studies in the United Kingdom, the Oxford Vegetarian Study (OVS) (10) and the EPIC-Oxford cohort (6), and included more than 5000 deaths before age 90. Thus, the present analysis mirrors a recent study of cancer incidence by diet group that used pooled data from the same 2 cohorts (11), while updating and extending the results from previous analyses of comparative mortality in United Kingdom-based vegetarians and nonvegetarians (8, 12).

## METHODS

### Recruitment of subjects

Participants in the OVS were recruited throughout the United Kingdom between 1980 and 1984 (10). Vegetarian participants were recruited through advertisements, the news media, and word of mouth. Nonvegetarian participants were recruited as friends and relatives of the vegetarian participants. In total, 11,140 subjects were recruited. At recruitment, participants completed a questionnaire on their diet and other lifestyle factors, including 4 questions on whether or not they consumed meat, fish, dairy products, and eggs, and 2 questions on the frequency of meat consumption.

The EPIC-Oxford cohort was recruited throughout the United Kingdom between 1993 and 1999 (6). A multicenter research ethics committee (Scotland A Research Ethics Committee) approved the protocol. Two methods of recruitment were used: general practice (GP) recruitment and postal recruitment, as described elsewhere (6). In total, 7,421 participants were recruited by the GP method and 57,990 participants by the postal method. The recruitment questionnaire, which can be viewed online at http://www.epic-oxford.org, included 4 questions on whether or not participants consumed meat, fish, dairy products, and eggs, and questions on the frequency of meat consumption. Surviving participants were sent follow-up questionnaires approximately 5, 10, and 15 y after recruitment, including the same 4 questions on current intake of meat, fish, dairy products, and eggs and ≥1 questions on the frequency of meat consumption, enabling us to classify them according to diet group at each time point. The 5 y, 10 y, and 15 y follow-up questionnaires can also be viewed online at http://www.epic-oxford.org.

For both studies, answers to the 4 questions on the consumption of meat, fish, dairy products, and eggs were used to assign participants to 1 of 4 diet groups at each time point [OVS recruitment, EPIC-Oxford recruitment, EPIC-Oxford follow-up questionnaire 1 (FU1), EPIC-Oxford follow-up questionnaire 2 (FU2), and EPIC-Oxford follow-up questionnaire 3 (FU3), as applicable]: meat eaters (participants who ate meat, irrespective of whether they ate fish, dairy products, or eggs), fish eaters (participants who did not eat meat but did eat fish), vegetarians (participants who did not eat meat or fish, but did eat either or both dairy products and eggs), and vegans (participants who did not eat meat, fish, eggs, or dairy products). Combining this information with data on the frequency of meat consumption enabled us to divide the meat eaters into regular meat eaters (who reported eating meat on ≥5 occasions per week on average) and low meat eaters (who ate meat <5 times/wk).

Participants in both studies were followed until the censoring date of 31 March 2014 by record linkage with the United Kingdom’s National Health Service Central Register, which provides information on deaths and their causes. Person-years were calculated from the beginning of recruitment until the date of death, emigration, or loss to follow-up, the participant’s 90th birthday, or the censoring date, whichever occurred first. Participants in the OVS who subsequently joined the EPIC-Oxford study contributed person-years in the OVS until the date when they joined the EPIC-Oxford study. For EPIC-Oxford participants, the person-years were split into 1 to 4 phases depending on which, if any, of the follow-up questionnaires they completed. Diet group and categories of each of the adjustment and stratification variables were reset as appropriate at the beginning of each phase. For example, a participant who joined the OVS as a regular meat eater and had become a low meat eater at recruitment to the EPIC-Oxford study and a fish eater at FU1 completion would contribute person-years to each of these diet groups.

The 18 common underlying causes of death for which HRs were calculated were as follows: malignant cancer [International Classification of Diseases (ICD)-10 codes C00–97 and equivalent ICD-9 codes], including colorectal cancer (ICD-10 C18–20), pancreatic cancer (C25), lung cancer (C34), female breast cancer (C50), ovarian cancer (C56), and cancers of the lymphatic/hematopoietic tissue (C81–96); mental and behavioral disorders (F00–99); diseases of the nervous system (G00–99); circulatory disease (I00–99), including ischemic heart disease (IHD) (I20–25), cerebrovascular disease (I60–69), and other circulatory disease (I00–15, I26–52, and I70–99); diseases of the respiratory system (J00–99); diseases of the digestive system (K00–93); injury, poisoning and external causes (S00–T98 and V01–Y98); all other causes (ICD-10 codes beginning with A, B, D, E, H, and L–R); and all causes combined.

### Statistical analysis

Participants were excluded from the analysis if they were aged <20 or >89 y at recruitment, or had a previous (registered or self-reported) malignant neoplasm before recruitment; a previous self-reported stroke, heart attack, or angina; uncertain follow-up; or had no information for ≥1 of the factors age, sex, smoking, and diet group at recruitment. EPIC-Oxford participants who did not complete the main questionnaire were also excluded because data on several important factors were thereby unavailable. After these exclusions, there were 60,310 participants (14,916 men and 45,394 women), including 10,359 OVS participants and 52,659 EPIC-Oxford participants; 2,708 participants contributed follow-up data from both studies. HRs (95% CIs) for 18 common causes of death, including all causes combined, were calculated by Cox proportional hazards regression with age as the underlying time variable, with the use of a clustered sandwich variance estimator to allow for intraparticipant correlation among individuals contributing person-years to >1 of the 5 possible phases of follow-up, including OVS recruitment to the earlier of EPIC-Oxford recruitment (if applicable) or death/censoring, EPIC-Oxford recruitment to the earliest of FU1/FU2/FU3 completion (if applicable) or death/censoring, FU1 completion (if applicable) to the earliest of FU2/FU3 completion (if applicable) or death/censoring, FU2 completion (if applicable) to the earlier of FU3 completion (if applicable) or death/censoring, and FU3 completion (if applicable) to death/censoring. For the small number of participants whose diet group was unknown at FU1, FU2, or FU3 completion (∼30 participants at each stage), diet group was deemed to be the same as at EPIC-Oxford recruitment or at the most-recently completed follow-up questionnaire, as appropriate. The analyses were stratified by study protocol (OVS participants, EPIC-Oxford GP-recruited participants, or EPIC-Oxford postal recruited participants), a 28-category variable combining sex; parity; oral contraceptive and hormone therapy use in women (men, plus the 27 combinations of nulliparous, parous, and unknown parity; never user, past or current user, and unknown user of oral contraceptives; and never user, past or current user, and unknown user of hormone therapy); self-reported prior diabetes (yes, no, or unknown); self-reported prior high blood pressure (yes, no, or unknown); and self-reported long-term medical treatment (yes, no, or unknown). HRs also were adjusted for smoking (never smoker; former smoker; current smoker of 1–9, 10–19, or ≥20 cigarettes/d; other current smoker, including pipe and cigar smokers; or unknown); alcohol consumption (<1, 1–7, 8–15, or ≥16 g ethanol/d or unknown); physical activity [low, high, or unknown; for OVS, high means sport/keep fit and/or running/cycling ≥2 times/wk, and low means neither of these (where known); for EPIC-Oxford, low means an average of <3.5 h/wk cycling or other physical exercise, and high means more than this (where known)]; marital status (married or cohabiting, not married or cohabiting, or unknown); and regular use of nutritional supplements (yes, no, or unknown), with optional further adjustment for BMI (in kg/m^2^; <18, 18–19.9, 20–21.9, 22–23.9, 24–25.9, 26–27.9, 28–29.9, 30–32.4, or ≥32.5, or unknown).

In the main analysis of mortality before age 90 y, the meat eaters were divided into 2 categories according to the average frequency of meat consumption, whereas the vegetarians and vegans were combined into a single group, creating 4 diet groups: regular meat eaters (who reported eating meat ≥5 times/wk on average), low meat eaters (who ate meat but did so <5 times/wk), fish eaters, and vegetarians and vegans. For the 6 most common causes of death (malignant cancer, circulatory disease, IHD, cerebrovascular disease, diseases of the respiratory system, and all causes combined), subgroup analyses were also conducted for men; women; participants with BMI <20 (underweight), BMI 20–24.9 (normal weight), and BMI ≥25 (overweight); never smokers, former smokers and current smokers; and after excluding the first 2 y of follow-up in either the OVS or EPIC-Oxford study according to which study participants joined first. We also conducted the mortality analyses for all 18 causes of death after excluding participants known to have changed diet group at least once during follow-up, including participants common to the OVS and EPIC-Oxford study who were in a different diet group at recruitment to the 2 studies. For the 6 most common causes of death only, we also calculated HRs with the vegetarians and vegans separated to give results for 5 diet groups (regular meat eaters, low meat eaters, fish eaters, vegetarians, and vegans). We also examined mortality before age 75 y in the model for the 4 diet groups for 17 causes of death (there were too few deaths from mental and behavioral disorders before age 75 for meaningful analysis), censoring at participants’ 75th birthday if this preceded their censoring date or date of death, emigration, or other loss to follow-up, repeating this analysis after excluding participants known to have changed diet group at least once during follow-up. The main results were not adjusted for BMI because we consider that the differences in BMI between the dietary groups are largely caused by the differences in diet, and, therefore, that BMI may mediate some of the differences in risk between dietary groups, but we do report the effects on the HR of further adjustment for BMI (in 10 categories, including unknown, as listed above).

Chi-square tests of heterogeneity of risk between the diet groups were based on the statistical significance of diet group in the model, the null hypothesis being equality of risk across the 4 (or 5) diet groups. Tests of heterogeneity of risks by diet group between men and women and categories of BMI and smoking were based on the statistical significance of the corresponding interaction term in the model. Statistical significance was set at the 5% level. All statistical analyses were conducted with the use of Stata Statistical Software: Release 14.

## RESULTS

The characteristics of the participants in each of 4 diet groups (regular meat eaters, low meat eaters, fish eaters, and vegetarians and vegans combined) are shown in **Table 1**. To avoid double counting, persons who participated in both the OVS and EPIC-Oxford study are grouped according to their characteristics at recruitment to the EPIC-Oxford study. One-third of participants were vegetarian or vegan and three-quarters were women. Two-thirds of the vegetarians and vegans had followed their diet for more than 5 y (results not shown), and 59% of meat eaters ate meat ≥5 times/wk on average. Mean age at recruitment was lower in the fish eaters than in the meat eaters, and lower still in the vegetarians and vegans. Smoking rates were low overall, with only 16% of men and 12% of women reporting that they were smokers at the time of recruitment. Mean BMI was ∼2 kg/m^2^ lower in vegetarians and vegans than in regular meat eaters and the proportion with a BMI <20 was 2–3 times as high in the former group as in the latter. Fish eaters had a slightly higher mean BMI than did the vegetarians and vegans. Mean alcohol consumption was lowest in the vegetarians and vegans and highest in fish eaters (women) or regular meat eaters (men). The proportions of men and women who reported a relatively high amount of physical activity were highest in fish eaters and lowest in regular meat eaters. About two-thirds of participants were married or cohabiting at recruitment, the proportion being highest in regular meat eaters and lowest in vegetarians and vegans. The proportions of men and women who reported regular use of nutritional supplements were highest in fish eaters and lowest in regular meat eaters, with more than one-third of men and more than one-half of women taking supplements. The proportion of women who were nulliparous at recruitment was highest among vegetarians and vegans and lowest among regular meat eaters, and the proportion of women who had ever used oral contraceptives was highest in fish eaters and lowest in regular meat eaters. Less than 2% of participants reported a previous diagnosis of diabetes, the percentages being highest in regular meat eaters and lowest in vegetarians and vegans; a similar pattern was found for self-reported prior high blood pressure and long-term medical treatment, except that the percentages were closer to 10% and 20%, respectively.

**TABLE 1:** Baseline characteristics by sex and diet group^1^

Characteristics	Regular meat eaters	Low meat eaters	Fish eaters	Vegetarians or vegans	All participants
Men					
Participants, *n*	5035	2911	1590	5380	14,916
Age at recruitment, y	48.4 ± 14.3	47.0 ± 14.8	43.0 ± 13.4	40.7 ± 14.3	44.8 ± 14.7
Smoking					
Never smoker	45.9	49.2	54.3	56.6	51.3
Former smoker	34.1	33.8	30.4	30.2	32.2
Current smoker (1–9 cigarettes/d)	3.3	4.1	4.7	4.0	3.8
Current smoker (10–19 cigarettes/d)	4.1	3.8	3.8	3.1	3.7
Current smoker (≥20 cigarettes/d)	6.5	4.3	2.5	3.1	4.4
Other current smoker^2^	6.2	4.8	4.3	2.9	4.5
BMI^3^					
<20 kg/m^2^	4.3	7.3	8.6	12.3	8.2
20–24.9 kg/m^2^	52.3	59.9	65.7	63.2	59.1
≥25 kg/m^2^	40.4	30.0	22.6	20.9	29.4
Unknown	3.0	2.8	3.1	3.6	3.2
Mean, kg/m^2^	24.8 ± 3.3	23.9 ± 3.0	23.3 ± 3.1	23.0 ± 3.1	23.8 ± 3.3
Alcohol consumption					
<1 g/d	8.4	12.9	12.4	22.2	14.7
1–7 g/d	28.5	30.7	27.9	29.2	29.1
8–15 g/d	25.6	24.1	25.0	21.4	23.7
≥16 g/d	35.7	30.7	32.3	25.2	30.6
Unknown	1.9	1.5	2.5	2.0	1.9
Mean, g/d	16.7 ± 17.7	13.9 ± 15.0	15.5 ± 17.2	12.6 ± 16.7	14.5 ± 16.9
Physical activity					
Low	64.7	57.7	53.8	55.3	58.8
High	29.0	35.4	39.1	38.9	34.9
Unknown	6.3	6.8	7.2	5.8	6.3
Marital status					
Married or cohabiting	75.5	69.3	66.1	60.8	68.0
Not married or cohabiting	24.3	30.4	33.6	38.9	31.8
Unknown	0.2	0.3	0.3	0.3	0.3
Regularly take nutritional supplements					
No	65.9	57.6	53.5	58.9	60.4
Yes	32.6	40.4	44.4	39.5	37.9
Unknown	1.5	2.0	2.1	1.5	1.7
Prior diabetes					
No	94.6	95.2	95.6	96.1	95.4
Yes	2.3	1.5	1.1	1.0	1.6
Unknown	3.1	3.4	3.3	2.8	3.1
Prior high blood pressure					
No	85.7	87.5	89.6	92.0	88.7
Yes	12.0	9.7	8.1	5.5	8.8
Unknown	2.3	2.8	2.3	2.5	2.5
Receiving long-term medical treatment					
No	74.9	79.3	81.8	84.4	79.9
Yes	23.9	19.0	16.6	14.7	18.8
Unknown	1.2	1.7	1.6	0.9	1.2
Food/nutrient intake^4^					
Energy, MJ/d	9.66 ± 2.40	8.48 ± 2.42	8.92 ± 2.42	8.66 ± 2.41	8.99 ± 2.46
Protein, % energy	16.5 ± 2.7	15.0 ± 2.5	13.9 ± 2.2	13.0 ± 2.0	14.6 ± 2.8
Animal protein, % energy	11.0 ± 2.7	8.5 ± 2.6	6.8 ± 2.3	5.2 ± 2.1	7.9 ± 3.5
Plant protein, % energy	5.5 ± 1.1	6.5 ± 1.3	7.0 ± 1.4	7.8 ± 1.7	6.7 ± 1.7
Carbohydrate, % energy	45.3 ± 6.1	49.2 ± 6.2	49.6 ± 6.5	51.8 ± 7.0	48.9 ± 7.1
Total fat, % energy	32.9 ± 5.4	30.8 ± 5.9	31.2 ± 6.0	30.6 ± 6.4	31.5 ± 6.0
Saturated fat, % energy	12.4 ± 3.1	11.1 ± 3.3	10.7 ± 3.3	10.0 ± 3.6	11.1 ± 3.5
Dietary fiber,^5^ g/d	18.0 ± 6.4	19.3 ± 7.6	21.5 ± 7.5	22.9 ± 8.0	20.4 ± 7.6
Total meat, g/d	115 ± 47	36 ± 18	—	—	45 ± 58
Red meat, g/d	84 ± 43	23 ± 13	—	—	32 ± 45
Poultry meat, g/d	31 ± 23	13 ± 13	—	—	13 ± 20
Total processed meat, g/d	31 ± 21	9 ± 6	—	—	12 ± 18
Total fish, g/d	42 ± 28	39 ± 33	41 ± 35	—	26 ± 31
Oily fish, g/d	13 ± 17	15 ± 21	16 ± 21	—	9 ± 16
Fresh fruit, g/d	200 ± 164	252 ± 219	248 ± 202	256 ± 227	236 ± 205
Fresh vegetables, g/d	220 ± 115	229 ± 133	263 ± 138	275 ± 146	247 ± 135
Women					
Participants, *n*	13,396	10,128	6926	14,944	45,394
Age at recruitment, y	47.7 ± 12.8	46.3 ± 13.7	40.7 ± 13.0	37.8 ± 13.7	43.0 ± 14.0
Smoking					
Never smoker	59.8	61.0	60.4	64.5	61.7
Former smoker	26.5	27.5	29.4	25.1	26.7
Current smoker (1–9 cigarettes/d)	3.6	4.2	4.4	4.5	4.2
Current smoker (10–19 cigarettes/d)	4.8	3.7	3.2	3.3	3.8
Current smoker (≥20 cigarettes/d)	4.6	2.8	1.9	1.9	2.9
Other current smoker^2^	0.7	0.8	0.7	0.7	0.7
BMI^3^					
<20 kg/m^2^	8.3	13.2	17.4	20.8	14.9
20–24.9 kg/m^2^	52.7	59.1	61.4	59.2	57.6
≥25 kg/m^2^	36.2	24.9	18.0	16.3	24.3
Unknown	2.8	2.9	3.1	3.7	3.2
Mean, kg/m^2^	24.7 ± 4.4	23.4 ± 3.7	22.7 ± 3.4	22.5 ± 3.4	23.4 ± 3.9
Alcohol consumption					
<1 g/d	16.5	18.6	16.8	24.4	19.6
1–7 g/d	46.7	45.7	42.9	42.0	44.4
8–15 g/d	22.6	23.0	24.7	21.2	22.5
≥16 g/d	11.6	11.2	13.7	11.0	11.6
Unknown	2.7	1.4	1.9	1.4	1.9
Mean, g/d	7.7 ± 9.4	7.5 ± 9.3	8.4 ± 10.1	7.2 ± 9.6	7.6 ± 9.6
Physical activity					
Low	68.0	62.0	57.1	59.9	62.4
High	20.9	26.6	32.2	31.1	27.3
Unknown	11.1	11.3	10.7	9.0	10.4
Marital status					
Married or cohabiting	76.1	63.0	62.5	58.3	65.2
Not married or cohabiting	23.8	36.8	37.3	41.3	34.5
Unknown	0.2	0.2	0.2	0.4	0.2
Regularly take nutritional supplements					
No	48.3	38.2	34.4	42.8	42.1
Yes	49.7	59.5	63.3	55.3	55.8
Unknown	1.9	2.3	2.3	1.9	2.1
Prior diabetes					
No	82.3	89.1	95.0	94.8	89.8
Yes	1.5	1.0	0.8	0.6	1.0
Unknown	16.3	9.9	4.2	4.7	9.2
Prior high blood pressure					
No	73.0	80.4	88.8	89.6	82.5
Yes	13.1	11.1	7.5	6.2	9.5
Unknown	13.9	8.5	3.8	4.2	7.9
Receiving long-term medical treatment					
No	61.3	69.6	77.3	80.9	72.0
Yes	25.6	24.5	21.3	17.8	22.1
Unknown	13.1	5.9	1.5	1.4	5.9
Parity					
Nulliparous	22.0	34.1	47.1	57.1	40.1
Parous	77.2	64.7	51.8	41.5	58.8
Unknown	0.8	1.2	1.1	1.4	1.1
Ever used oral contraceptives					
No	29.8	28.9	21.1	24.6	26.6
Yes	68.8	70.1	78.5	74.8	72.5
Unknown	1.3	1.0	0.4	0.6	0.9
Ever used hormone therapy					
No	63.8	69.0	80.9	78.7	72.5
Yes	26.0	20.0	10.8	6.7	16.0
Unknown	10.2	10.9	8.2	14.6	11.5
Food/nutrient intake^4^					
Energy, MJ/d	8.45 ± 2.07	7.44 ± 2.01	7.73 ± 2.10	7.54 ± 2.10	7.82 ± 2.11
Protein, % energy	18.2 ± 2.9	16.2 ± 2.7	14.8 ± 2.3	13.8 ± 2.1	15.8 ± 3.1
Animal protein, % energy	12.3 ± 2.9	9.5 ± 2.7	7.6 ± 2.4	5.9 ± 2.2	8.9 ± 3.7
Plant protein, % energy	5.8 ± 1.1	6.6 ± 1.3	7.2 ± 1.4	7.8 ± 1.7	6.9 ± 1.6
Carbohydrate, % energy	46.8 ± 5.6	50.3 ± 6.2	51.2 ± 6.4	53.2 ± 6.7	50.3 ± 6.7
Total fat, % energy	32.3 ± 5.6	30.5 ± 6.2	30.7 ± 6.3	30.1 ± 6.7	30.9 ± 6.3
Saturated fat, % energy	11.9 ± 3.1	10.9 ± 3.4	10.6 ± 3.3	10.2 ± 3.5	10.9 ± 3.4
Dietary fiber,^5^ g/d	18.4 ± 6.3	19.2 ± 7.2	21.1 ± 7.4	21.8 ± 8.1	20.1 ± 7.4
Total meat, g/d	106 ± 40	35 ± 18	—	—	40 ± 51
Red meat, g/d	70 ± 36	21 ± 12	—	—	26 ± 36
Poultry meat, g/d	36 ± 23	15 ± 14	—	—	14 ± 21
Total processed meat, g/d	24 ± 16	8 ± 5	—	—	9 ± 14
Total fish, g/d	44 ± 28	41 ± 30	38 ± 33	—	28 ± 31
Oily fish, g/d	15 ± 17	16 ± 17	16 ± 19	—	11 ± 16
Fresh fruit, g/d	257 ± 189	298 ± 231	299 ± 230	293 ± 244	285 ± 224
Fresh vegetables, g/d	256 ± 125	262 ± 146	292 ± 151	301 ± 169	277 ± 150

1 Values are means ± SDs or percentages. Persons who participated in both the Oxford Vegetarian Study and EPIC-Oxford are grouped according to their characteristics at recruitment to EPIC-Oxford. EPIC-Oxford, European Prospective Investigation into Cancer and Nutrition–Oxford.

2 Includes pipe or cigar smokers and current smokers of an unknown number of cigarettes per day.

3 Known for 14,441 men and 43,951 women.

4 In 11,625 men and 39,936 women in the EPIC-Oxford study with reliable nutrient intake data.

5 As a nonstarch polysaccharide.

The estimated mean intake of selected foods and nutrients by diet group is also shown in Table 1. Because the OVS recruitment questionnaire was insufficiently detailed to enable nutrient intake to be estimated, the values are based on data from the EPIC-Oxford study only. Expressed as a percentage of energy intake, intake of protein, total fat, and saturated fat was highest in regular meat eaters and lowest in vegetarians and vegans, whereas the reverse was true for intake of carbohydrate, dietary fiber, and plant protein. Meat intake was >3 times higher in regular meat eaters than in low meat eaters (∼110 g/d and ∼35 g/d, respectively), whereas total fish intake was ∼40 g/d in each of the nonvegetarian groups, about one-third of which was oily fish. Intake of fresh fruit and vegetables was lowest in regular meat eaters and generally highest in vegetarians and vegans, although the differences between the highest and lowest mean intake was only ∼50 g/d.

Of the 2708 persons who participated in both the OVS and EPIC-Oxford study, 1839 (68%) were allocated to the same diet group (regular meat eater, low meat eater, fish eater, or vegetarian or vegan) at recruitment to both studies, with an average 13 y gap between recruitment dates, indicating a high degree of consistency in diet group. Of the 52,659 EPIC-Oxford participants included in the analysis, 34,983 completed FU1 ∼5 y after recruitment and could be characterized according to diet group at this time. Of these, 25,555 (73%) were allocated to the same diet group (regular meat eater, low meat eater, fish eater, or vegetarian or vegan) as they had been at recruitment.

There were 5294 deaths before age 90 among the participants in >1 million years of follow-up. **Table 2** shows the HR for low meat eaters, fish eaters, and vegetarians and vegans combined relative to regular meat eaters for the 18 causes of death investigated, each of which was responsible for more than 130 deaths before age 90, without and with additional adjustment for BMI. For all causes of death combined, there was no significant difference in risk between diet groups [low meat eaters, HR: 0.93 (95% CI: 0.86, 1.00); fish eaters, HR: 0.96 (95% CI: 0.86, 1.06); and vegetarians and vegans, HR: 1.02 (95% CI: 0.94, 1.10) compared with regular meat eaters; *P*-heterogeneity of risks = 0.082]. There was significant heterogeneity of risk between diet groups for deaths from pancreatic cancer [low meat eaters, HR: 0.55 (95% CI: 0.36, 0.86) and vegetarians and vegans, HR: 0.48 (95% CI 0.28, 0.82), compared with regular meat eaters; *P*-heterogeneity = 0.012] and cancers of the lymphatic/hematopoietic tissue [vegetarians and vegans compared with regular meat eaters, HR: 0.50 (95% CI: 0.32, 0.79);* P*-heterogeneity = 0.010]. Mortality from all malignant cancers combined was significantly lower in fish eaters than in regular meat eaters, HR: 0.82 (95% CI: 0.70, 0.97). There was also significant heterogeneity of risk between diet groups for circulatory disease mortality [fish eaters compared with regular meat eaters, HR: 1.22 (95% CI: 1.02, 1.46); *P*-heterogeneity = 0.046]; cerebrovascular disease mortality (*P*-heterogeneity = 0.023, but no significant differences in risk between regular meat eaters and any of the other diet groups); and respiratory disease mortality [low meat eaters compared with regular meat eaters, HR: 0.70 (95% CI: 0.53, 0.92);* P*-heterogeneity = 0.020]. Mortality from all other causes (ICD-10 codes beginning with A, B, D, E, H, and L–R) was significantly lower in low meat eaters than in regular meat eaters (HR: 0.74 (95% CI: 0.56, 0.99), although there was no overall heterogeneity of risk between the diet groups for this endpoint (*P*-heterogeneity = 0.13). Further adjustment for BMI left these associations largely unchanged, except that the higher mortality from other circulatory disease in fish eaters compared with regular meat eaters became statistically significant (HR: 1.45 (95% CI: 1.03, 2.03); *P*-heterogeneity = 0.20).

**TABLE 2:** Number of deaths before age 90 y and HRs (95% CIs) by diet group^1^

	Regular meat eaters		Low meat eaters		Fish eaters		Vegetarians and vegans		
Cause of death (ICD-10 codes) for each model	*n*	HR	*n*	HR (95% CI)	*n*	HR (95% CI)	*n*	HR (95% CI)	*P*-het^2^
Malignant cancer (C00–97)	819		593		205		520		
Basic		1.00		0.96 (0.86, 1.07)		0.82 (0.70, 0.97)		0.93 (0.82, 1.05)	0.12
+BMI		1.00		0.96 (0.86, 1.07)		0.81 (0.69, 0.95)		0.91 (0.80, 1.03)	0.074
Colorectum (C18–20)	97		71		21		76		
Basic		1.00		0.99 (0.72, 1.35)		0.73 (0.45, 1.21)		1.13 (0.80, 1.59)	0.41
+BMI		1.00		0.99 (0.72, 1.35)		0.72 (0.44, 1.18)		1.11 (0.79, 1.58)	0.41
Pancreas (C25)	69		30		14		20		
Basic		1.00		0.55 (0.36, 0.86)		0.70 (0.39, 1.25)		0.48 (0.28, 0.82)	0.012
+BMI		1.00		0.54 (0.35, 0.85)		0.66 (0.37, 1.19)		0.44 (0.26, 0.76)	0.006
Lung (C34)	110		64		15		62		
Basic		1.00		0.85 (0.62, 1.17)		0.60 (0.34, 1.06)		1.14 (0.80, 1.62)	0.12
+BMI		1.00		0.82 (0.60, 1.14)		0.56 (0.32, 1.00)		1.07 (0.75, 1.54)	0.11
Female breast (C50)	75		69		35		70		
Basic		1.00		1.10 (0.78, 1.54)		1.19 (0.77, 1.83)		1.13 (0.78, 1.63)	0.86
+BMI		1.00		1.09 (0.78, 1.52)		1.19 (0.78, 1.84)		1.12 (0.77, 1.63)	0.87
Ovary (C56)	63		44		15		41		
Basic		1.00		0.91 (0.61, 1.37)		0.69 (0.37, 1.28)		0.97 (0.63, 1.49)	0.68
+BMI		1.00		0.91 (0.61, 1.37)		0.68 (0.37, 1.28)		0.97 (0.61, 1.52)	0.67
Lymphatic/hematopoietic tissue (C81–96)	85		63		27		28		
Basic		1.00		0.98 (0.70, 1.38)		1.09 (0.68, 1.73)		0.50 (0.32, 0.79)	0.010
+BMI		1.00		0.95 (0.67, 1.34)		1.03 (0.64, 1.64)		0.47 (0.30, 0.73)	0.004
Mental and behavioral disorders (F00–99)	45		36		15		50		
Basic		1.00		0.88 (0.54, 1.42)		0.92 (0.48, 1.75)		1.22 (0.78, 1.91)	0.53
+BMI		1.00		0.84 (0.52, 1.36)		0.86 (0.45, 1.62)		1.12 (0.71, 1.77)	0.65
Diseases of the nervous system (G00–99)	78		67		24		57		
Basic		1.00		0.97 (0.68, 1.38)		0.99 (0.61, 1.59)		0.95 (0.64, 1.41)	1.00
+BMI		1.00		0.94 (0.66, 1.35)		0.89 (0.54, 1.47)		0.83 (0.54, 1.26)	0.85
Circulatory disease (I00–99)	542		391		178		433		
Basic		1.00		0.96 (0.84, 1.10)		1.22 (1.02, 1.46)		1.10 (0.95, 1.27)	0.046
+BMI		1.00		0.98 (0.85, 1.13)		1.26 (1.05, 1.51)		1.13 (0.97, 1.30)	0.028
Ischemic heart disease (I20–25)	245		162		62		175		
Basic		1.00		0.93 (0.76, 1.15)		1.00 (0.75, 1.34)		0.99 (0.79, 1.23)	0.93
+BMI		1.00		0.96 (0.78, 1.19)		1.06 (0.80, 1.42)		1.03 (0.82, 1.28)	0.92
Cerebrovascular disease (I60–69)	162		116		62		152		
Basic		1.00		0.87 (0.68, 1.13)		1.35 (0.99, 1.85)		1.21 (0.94, 1.56)	0.023
+BMI		1.00		0.88 (0.68, 1.13)		1.35 (0.98, 1.86)		1.19 (0.91, 1.54)	0.034
Other circulatory disease (I00–15, I26–52, and I70–99)	135		113		54		106		
Basic		1.00		1.10 (0.85, 1.43)		1.39 (0.99, 1.94)		1.16 (0.87, 1.54)	0.29
+BMI		1.00		1.14 (0.88, 1.47)		1.45 (1.03, 2.03)		1.20 (0.89, 1.61)	0.20
Diseases of the respiratory system (J00–99)	166		93		39		131		
Basic		1.00		0.70 (0.53, 0.92)		0.95 (0.65, 1.37)		1.09 (0.83, 1.42)	0.020
+BMI		1.00		0.70 (0.53, 0.92)		0.88 (0.60, 1.28)		1.02 (0.77, 1.35)	0.041
Diseases of the digestive system (K00–93)	77		61		18		54		
Basic		1.00		1.10 (0.78, 1.55)		0.74 (0.43, 1.26)		0.94 (0.64, 1.39)	0.54
+BMI		1.00		1.10 (0.78, 1.55)		0.73 (0.43, 1.24)		0.94 (0.63, 1.39)	0.52
Injury, poisoning, and external causes (S00-T98 and V01-Y98)	66		50		32		83		
Basic		1.00		0.91 (0.63, 1.31)		1.09 (0.70, 1.70)		1.09 (0.77, 1.54)	0.79
+BMI		1.00		0.87 (0.60, 1.26)		0.99 (0.63, 1.55)		0.96 (0.68, 1.37)	0.90
All other causes (codes beginning with A, B, D, E, H, or L–R)	136		84		39		112		
Basic		1.00		0.74 (0.56, 0.99)		0.85 (0.58, 1.25)		1.02 (0.77, 1.35)	0.13
+BMI		1.00		0.73 (0.55, 0.98)		0.83 (0.56, 1.22)		0.96 (0.71, 1.28)	0.16
All causes (A00–Y98)	1929		1375		550		1440		
Basic		1.00		0.93 (0.86, 1.00)		0.96 (0.86, 1.06)		1.02 (0.94, 1.10)	0.082
+BMI		1.00		0.93 (0.86, 1.00)		0.94 (0.85, 1.04)		0.99 (0.92, 1.07)	0.15

1 Estimated by Cox proportional hazards regression with age as the underlying time variable. Basic model adjusted for smoking (never smoker; former smoker; current smoker of 1–9, 10–19, or ≥20 cigarettes per day; other current smoker; unknown); alcohol consumption (<1, 1–7, 8–15, or ≥16 g ethanol/d or unknown); physical activity (low, high, or unknown); whether married or cohabiting (yes, no, or unknown); and regular use of nutritional supplements (no, yes, or unknown), and stratified by study/method of recruitment (Oxford Vegetarian Study, EPIC-Oxford postal, or EPIC-Oxford general practice); all possible combinations of sex, parity (nulliparous, parous, or unknown), oral contraceptive use, and hormone therapy use (both ever, never, or unknown); prior diabetes; prior high blood pressure; and receipt of long-term medical treatment (each no, yes, or unknown), with the use of separate models for each endpoint. Model +BMI is further adjusted for BMI (in kg/m^2^; <18, 18.0–19.9, 20.0–21.9, 22.0–23.9, 24.0–25.9, 26.0–27.9, 28.0–29.9, 30.0–32.4, or ≥32.5, or unknown). EPIC-Oxford, European Prospective Investigation into Cancer and Nutrition–Oxford; het, heterogeneity; ICD, International Classification of Diseases.

2 Chi-square test of heterogeneity of risk between the 4 diet groups.

With 2 exceptions, there was no significant heterogeneity of risks between men and women; between never, former, and current smokers; or between low-weight, normal-weight, and overweight participants for any of the 6 most common causes of death (malignant cancer, circulatory disease, IHD, cerebrovascular disease, diseases of the respiratory system, and all causes combined) for which subgroup analyses were performed (results not shown). The exceptions were circulatory disease mortality subdivided by BMI category (*P*-interaction = 0.030), for which there was significant heterogeneity of risks between diet groups for participants with BMI ≥25 [fish eaters, HR: 1.53 (95% CI: 1.13, 2.08), and vegetarians and vegans, HR: 1.28 (95% CI: 1.00, 1.64) compared with regular meat eaters; *P*-heterogeneity = 0.015] but not for participants with BMI <20 or 20–24.9; and respiratory disease mortality subdivided by smoking status (*P*-interaction = 0.047), for which there was significant heterogeneity of risks between diet groups for never smokers [vegetarians and vegans compared with regular meat eaters, HR: 1.53 (95% CI: 1.00, 2.37); *P*-heterogeneity = 0.024], but not for former smokers or current smokers.

When the first 2 y of follow-up were excluded, leaving 5133 deaths before age 90, there was significant heterogeneity of risk between the diet groups for all causes of death combined, as follows: low meat eaters, HR: 0.94 (95% CI: 0.87, 1.01); fish eaters, HR: 0.96 (95% CI: 0.86, 1.06); and vegetarians and vegans, HR: 1.05 (95% CI: 0.97, 1.13) compared with regular meat eaters; *P*-heterogeneity = 0.035. The heterogeneity of risks for circulatory disease, cerebrovascular disease and respiratory disease mortality all remained statistically significant (*P*-heterogeneity = 0.040, 0.021, and 0.016, respectively; HRs not shown). Further adjustment for BMI left these associations largely unchanged except that the heterogeneity of risk between the diet groups for all causes of death after excluding the first 2 y of follow-up was no longer statistically significant (*P*-heterogeneity = 0.10; HRs not shown).

When we excluded data for participants known to have changed diet group at least once during follow-up, leaving data for 4270 deaths before age 90, there was no significant difference in risk between diet groups for all causes of death combined, as follows: low meat eaters, HR: 0.93 (95% CI: 0.85, 1.02); fish eaters, HR: 0.91 (95% CI: 0.81, 1.02); and vegetarians and vegans, HR: 0.92 (95% CI: 0.84, 0.99) compared with regular meat eaters; *P*-heterogeneity = 0.13 (**Table 3**). There was significant heterogeneity of risk between the diet groups for mortality from all malignant cancers combined and cancers of the lymphatic/hematopoietic tissue (*P*-heterogeneity = 0.006 and 0.001, respectively). For all malignant cancers, both fish eaters and vegetarians and vegans combined had significantly lower mortality than regular meat eaters [HR: 0.76 (95% CI: 0.63, 0.91) and HR: 0.82 (95% CI: 0.72, 0.94), respectively]. Vegetarians and vegans combined also had significantly lower mortality than did regular meat eaters for pancreatic cancer [HR: 0.47 (95% CI: 0.26, 0.86); *P*-heterogeneity = 0.065] and cancers of the lymphatic/hematopoietic tissue [HR: 0.43 (95% CI: 0.27, 0.70)], and low meat eaters had significantly lower respiratory disease mortality than regular meat eaters [HR: 0.69 (95% CI: 0.49, 0.97); *P*-heterogeneity = 0.14]. Further adjustment for BMI left these associations largely unchanged except that the heterogeneity of risk between the diet groups for deaths from pancreatic cancer became statistically significant (*P*-heterogeneity = 0.029), with low meat eaters and vegetarians and vegans both having significantly lower pancreatic cancer mortality than regular meat eaters [HR: 0.55 (95% CI: 0.30, 0.98) and HR: 0.42 (95% CI: 0.23, 0.77), respectively].

**TABLE 3:** Number of deaths before age 90 y and HRs (95% CIs) by diet group after excluding data for participants known to have changed diet group at least once during follow-up^1^

	Regular meat eaters		Low meat eaters		Fish eaters		Vegetarians and vegans		
Cause of death (ICD-10 codes) for each model	*n*	HR	*n*	HR (95% CI)	*n*	HR (95% CI)	*n*	HR (95% CI)	*P*-het^2^
Malignant cancer (C00–97)	686		361		151		503		
Basic		1.00		0.93 (0.81, 1.07)		0.76 (0.63, 0.91)		0.82 (0.72, 0.94)	0.006
+BMI		1.00		0.93 (0.81, 1.06)		0.75 (0.62, 0.90)		0.81 (0.71, 0.93)	0.003
Colorectum (C18–20)	80		42		14		75		
Basic		1.00		0.95 (0.64, 1.42)		0.61 (0.33, 1.14)		1.05 (0.72, 1.53)	0.37
+BMI		1.00		0.96 (0.64, 1.44)		0.61 (0.33, 1.14)		1.05 (0.72, 1.54)	0.36
Pancreas (C25)	54		18		11		19		
Basic		1.00		0.57 (0.32, 1.02)		0.73 (0.37, 1.44)		0.47 (0.26, 0.86)	0.065
+BMI		1.00		0.55 (0.30, 0.98)		0.67 (0.34, 1.31)		0.42 (0.23, 0.77)	0.029
Lung (C34)	96		41		12		58		
Basic		1.00		0.92 (0.63, 1.33)		0.61 (0.32, 1.16)		1.06 (0.73, 1.55)	0.37
+BMI		1.00		0.88 (0.61, 1.29)		0.57 (0.29, 1.11)		1.00 (0.67, 1.47)	0.36
Female breast (C50)	63		39		25		67		
Basic		1.00		0.95 (0.62, 1.45)		1.08 (0.65, 1.79)		1.00 (0.67, 1.49)	0.97
+BMI		1.00		0.95 (0.62, 1.46)		1.11 (0.67, 1.85)		1.02 (0.68, 1.54)	0.96
Ovary (C56)	52		27		10		40		
Basic		1.00		0.89 (0.54, 1.45)		0.59 (0.28, 1.26)		0.85 (0.53, 1.36)	0.59
+BMI		1.00		0.88 (0.53, 1.45)		0.58 (0.28, 1.24)		0.83 (0.50, 1.38)	0.58
Lymphatic/hematopoietic tissue (C81–96)	71		44		23		28		
Basic		1.00		1.08 (0.71, 1.63)		1.05 (0.62, 1.78)		0.43 (0.27, 0.70)	0.001
+BMI		1.00		1.02 (0.67, 1.56)		0.99 (0.58, 1.69)		0.40 (0.24, 0.65)	<0.001
Mental and behavioral disorders (F00–99)	34		16		11		49		
Basic		1.00		0.65 (0.34, 1.24)		1.00 (0.47, 2.12)		1.07 (0.65, 1.77)	0.49
+BMI		1.00		0.62 (0.32, 1.19)		0.94 (0.44, 2.01)		1.00 (0.60, 1.67)	0.49
Diseases of the nervous system (G00–99)	59		41		18		53		
Basic		1.00		1.06 (0.68, 1.64)		1.03 (0.58, 1.81)		0.85 (0.54, 1.34)	0.77
+BMI		1.00		1.03 (0.65, 1.62)		0.98 (0.54, 1.77)		0.79 (0.49, 1.26)	0.62
Circulatory disease (I00–99)	466		274		132		419		
Basic		1.00		1.02 (0.86, 1.19)		1.10 (0.89, 1.36)		1.00 (0.86, 1.16)	0.81
+BMI		1.00		1.05 (0.89, 1.23)		1.15 (0.93, 1.42)		1.03 (0.88, 1.20)	0.64
Ischemic heart disease (I20–25)	220		118		48		170		
Basic		1.00		1.00 (0.79, 1.28)		0.89 (0.63, 1.25)		0.88 (0.70, 1.11)	0.65
+BMI		1.00		1.05 (0.82, 1.33)		0.95 (0.67, 1.33)		0.93 (0.73, 1.17)	0.81
Cerebrovascular disease (I60–69)	136		80		45		148		
Basic		1.00		0.91 (0.67, 1.23)		1.23 (0.85, 1.79)		1.15 (0.87, 1.52)	0.29
+BMI		1.00		0.92 (0.67, 1.24)		1.25 (0.86, 1.83)		1.15 (0.87, 1.53)	0.30
Other circulatory disease (I00–15, I26–52, and I70–99)	110		76		39		101		
Basic		1.00		1.20 (0.88, 1.64)		1.31 (0.89, 1.93)		1.04 (0.76, 1.41)	0.42
+BMI		1.00		1.24 (0.90, 1.69)		1.38 (0.93, 2.04)		1.08 (0.79, 1.48)	0.32
Diseases of the respiratory system (J00–99)	145		57		27		126		
Basic		1.00		0.69 (0.49, 0.97)		0.79 (0.50, 1.23)		0.95 (0.72, 1.27)	0.14
+BMI		1.00		0.70 (0.50, 0.98)		0.73 (0.47, 1.15)		0.91 (0.67, 1.22)	0.16
Diseases of the digestive system (K00–93)	64		32		14		49		
Basic		1.00		0.95 (0.62, 1.46)		0.79 (0.43, 1.46)		0.82 (0.53, 1.27)	0.78
+BMI		1.00		0.96 (0.62, 1.49)		0.80 (0.43, 1.49)		0.84 (0.53, 1.33)	0.85
Injury, poisoning, and external causes (S00–T98 and V01–Y98)	53		36		26		79		
Basic		1.00		1.07 (0.70, 1.65)		1.22 (0.74, 2.01)		1.03 (0.70, 1.52)	0.88
+BMI		1.00		1.04 (0.67, 1.60)		1.13 (0.69, 1.87)		0.94 (0.64, 1.39)	0.90
All other causes (codes beginning with A, B, D, E, H, or L–R)	102		53		29		105		
Basic		1.00		0.83 (0.58, 1.18)		1.01 (0.64, 1.60)		1.04 (0.76, 1.42)	0.64
+BMI		1.00		0.81 (0.56, 1.16)		0.95 (0.59, 1.53)		0.97 (0.69, 1.34)	0.69
All causes (A00–Y98)	1609		870		408		1383		
Basic		1.00		0.93 (0.85, 1.02)		0.91 (0.81, 1.02)		0.92 (0.84, 0.99)	0.13
+BMI		1.00		0.93 (0.85, 1.02)		0.90 (0.80, 1.01)		0.90 (0.83, 0.98)	0.079

1 Estimated by Cox proportional hazards regression with age as the underlying time variable. Basic model adjusted for smoking (never smoker; former smoker; current smoker of 1–9, 10–19, or ≥20 cigarettes per day; other current smoker; unknown); alcohol consumption (<1, 1–7, 8–15, or ≥16 g ethanol/d or unknown); physical activity (low, high, or unknown); whether married or cohabiting (yes, no, or unknown); and regular use of nutritional supplements (no, yes, or unknown), and stratified by study/method of recruitment (Oxford Vegetarian Study, EPIC-Oxford postal, or EPIC-Oxford general practice); all possible combinations of sex, parity (nulliparous, parous, or unknown), oral contraceptive use, and hormone therapy use (both ever, never, or unknown); prior diabetes; prior high blood pressure; and receipt of long-term medical treatment (each no, yes, or unknown), with the use of separate models for each endpoint. Model +BMI is further adjusted for BMI (in kg/m^2^; <18, 18.0–19.9, 20.0–21.9, 22.0–23.9, 24.0–25.9, 26.0–27.9, 28.0–29.9, 30.0–32.4, or ≥32.5, or unknown). EPIC-Oxford, European Prospective Investigation into Cancer and Nutrition–Oxford; het, heterogeneity; ICD, International Classification of Diseases.

2 Chi-square test of heterogeneity of risk between the 4 diet groups.

For 6 major causes of death (including all causes combined), vegetarians and vegans were separated and HRs compared with regular meat eaters were calculated for each of low meat eaters, fish eaters, vegetarians, and vegans (**Table 4**). For all causes of death, there was no significant difference in risk between diet groups as follows: low meat eaters, HR: 0.93 (95% CI: 0.86, 1.00); fish eaters, HR: 0.96 (95% CI: 0.87, 1.06); vegetarians, HR: 1.00 (95% CI: 0.93, 1.08); and vegans, HR: 1.14 (95% CI: 0.97, 1.35) compared with regular meat eaters; *P*-heterogeneity = 0.056. There was significant heterogeneity of risk between diet groups for cerebrovascular disease and respiratory disease mortality (*P*-heterogeneity = 0.023 and 0.015, respectively), with vegans having the highest mortality for both of these causes of death [compared with regular meat eaters, HR: 1.63 (95% CI: 0.98, 2.69) and HR: 1.57 (95% CI: 0.92, 2.67), respectively], but the CIs for the HRs in vegans were wide, precluding any clear conclusions. Further adjustment for BMI made little difference to the results. When we repeated this analysis after excluding data for participants known to have changed diet group at least once during follow-up, there was significant heterogeneity of risks between diet groups for malignant cancer mortality alone (*P*-heterogeneity = 0.015; results not shown). HRs for vegans compared with regular meat eaters for deaths from malignant cancer, circulatory disease, IHD, cerebrovascular disease, diseases of the respiratory system, and all causes combined were HR: 0.97 (95% CI: 0.72, 1.29); HR: 1.09 (95% CI: 0.76, 1.56); HR: 0.79 (95% CI: 0.44, 1.43); HR: 1.50 (95% CI: 0.84, 2.68); HR: 1.00 (95% CI: 0.50, 2.01); and HR: 1.00 (95% CI: 0.83, 1.20), respectively. Again, further adjustment for BMI made little difference to the results.

**TABLE 4:** Number of deaths before age 90 y and HRs (95% CIs) by diet group for common causes of death, showing separate results for vegetarians and vegans^1^

	Regular meat eaters		Low meat eaters		Fish eaters		Vegetarians		Vegans		
Cause of death (ICD-10 codes) for each model	*n*	HR	*n*	HR (95% CI)	*n*	HR (95% CI)	*n*	HR (95% CI)	*n*	HR (95% CI)	*P*-het^2^
Malignant cancer (C00–97)	819		593		205		453		67		
Basic		1.00		0.96 (0.87, 1.08)		0.83 (0.70, 0.97)		0.91 (0.80, 1.03)		1.14 (0.88, 1.47)	0.069
+BMI		1.00		0.96 (0.86, 1.07)		0.81 (0.69, 0.96)		0.89 (0.78, 1.01)		1.10 (0.85, 1.42)	0.053
Circulatory disease (I00–99)	542		391		178		390		43		
Basic		1.00		0.96 (0.84, 1.10)		1.22 (1.02, 1.46)		1.10 (0.95, 1.27)		1.16 (0.84, 1.59)	0.087
+BMI		1.00		0.98 (0.85, 1.13)		1.26 (1.05, 1.51)		1.12 (0.96, 1.30)		1.21 (0.88, 1.66)	0.053
Ischemic heart disease (I20–25)	245		162		62		161		14		
Basic		1.00		0.93 (0.76, 1.15)		1.00 (0.75, 1.34)		1.00 (0.80, 1.25)		0.85 (0.51, 1.44)	0.94
+BMI		1.00		0.96 (0.78, 1.19)		1.06 (0.79, 1.42)		1.04 (0.83, 1.30)		0.90 (0.53, 1.55)	0.94
Cerebrovascular disease (I60–69)	162		116		62		133		19		
Basic		1.00		0.88 (0.68, 1.13)		1.36 (0.99, 1.86)		1.17 (0.90, 1.51)		1.63 (0.98, 2.69)	0.023
+BMI		1.00		0.88 (0.68, 1.14)		1.36 (0.99, 1.87)		1.15 (0.88, 1.50)		1.61 (0.97, 2.69)	0.033
Diseases of the respiratory system (J00–99)	166		93		39		113		18		
Basic		1.00		0.70 (0.53, 0.92)		0.95 (0.65, 1.38)		1.04 (0.79, 1.37)		1.57 (0.92, 2.67)	0.015
+BMI		1.00		0.70 (0.53, 0.92)		0.89 (0.61, 1.29)		0.98 (0.73, 1.31)		1.48 (0.86, 2.56)	0.029
All causes (A00–Y98)	1929		1375		550		1274		166	
Basic		1.00		0.93 (0.86, 1.00)		0.96 (0.87, 1.06)		1.00 (0.93, 1.08)		1.14 (0.97, 1.35)	0.056
+BMI		1.00		0.93 (0.86, 1.00)		0.94 (0.85, 1.04)		0.98 (0.90, 1.06)		1.11 (0.94, 1.30)	0.11

1 Estimated by Cox proportional hazards regression with age as the underlying time variable. Basic model adjusted for smoking (never smoker; former smoker; current smoker of 1–9, 10–19, or ≥20 cigarettes per day; other current smoker; unknown); alcohol consumption (<1, 1–7, 8–15, or ≥16 g ethanol/d or unknown); physical activity (low, high, or unknown); whether married or cohabiting (yes, no, or unknown); and regular use of nutritional supplements (no, yes, or unknown), and stratified by study/method of recruitment (Oxford Vegetarian Study, EPIC-Oxford postal, or EPIC-Oxford general practice); all possible combinations of sex, parity (nulliparous, parous, or unknown), oral contraceptive use, and hormone therapy use (both ever, never, or unknown); prior diabetes; prior high blood pressure; and receipt of long-term medical treatment (each no, yes, or unknown), with the use of separate models for each endpoint. Model +BMI is further adjusted for BMI (in kg/m^2^; <18, 18.0–19.9, 20.0–21.9, 22.0–23.9, 24.0–25.9, 26.0–27.9, 28.0–29.9, 30.0–32.4, or ≥32.5, or unknown). EPIC-Oxford, European Prospective Investigation into Cancer and Nutrition–Oxford; het, heterogeneity; ICD, International Classification of Diseases.

2 Chi-square test of heterogeneity of risk between the 5 diet groups.

We also examined mortality before age 75 for the categorization with 4 diet groups including vegetarians and vegans combined. There were 2601 deaths before age 75 among the participants up to the censoring date. **T**he HRs for low meat eaters, fish eaters, and vegetarians and vegans combined relative to regular meat eaters for 17 causes of death are shown in **Supplemental Table 1** (mental and behavioral disorders were responsible for only 18 deaths before age 75—too few for meaningful analysis). For all-cause mortality before age 75, there was no significant difference in risk between diet groups: HRs for low meat eaters, fish eaters, and vegetarians and vegans compared with regular meat eaters were HR: 0.92 (95% CI: 0.83, 1.01); HR: 0.90 (95% CI: 0.78, 1.04); and HR: 0.97 (95% CI: 0.87, 1.08), respectively; *P*-heterogeneity = 0.28. Significant heterogeneity of risks between the diet groups was found for pancreatic cancer, lung cancer, and cancers of the lymphatic/hematopoietic tissue, and malignant cancer mortality was significantly lower in fish eaters than in regular meat eaters (Supplemental Table 1). Again, further adjustment for BMI made little difference to the results.

When we excluded data for participants known to have changed diet group at least once during follow-up from the early mortality analysis, leaving data for 2155 deaths before age 75, vegetarians and vegans had significantly lower all-cause mortality than regular meat eaters, although there was no overall heterogeneity of risk between the diet groups: HRs for low meat eaters, fish eaters, and vegetarians and vegans compared with regular meat eaters were HR: 0.97 (95% CI: 0.86, 1.09); HR: 0.87 (95% CI: 0.74, 1.02); and HR: 0.86 (95% CI: 0.77, 0.97), respectively; *P*-heterogeneity = 0.068 (**Supplemental Table 2**). There was significant heterogeneity of risk between the diet groups for mortality from all malignant cancers combined, cancers of the lung and lymphatic/hematopoietic tissue, and other circulatory disease. In addition, vegetarians and vegans had significantly lower mortality than regular meat eaters for pancreatic cancer and digestive diseases, and fish eaters had significantly lower colorectal cancer mortality than regular meat eaters (Supplemental Table 2). Further adjustment for BMI left these associations largely unchanged.

## DISCUSSION

In this analysis of mortality by diet group in a population with a high percentage of vegetarians and others who eat little or no meat, we found no significant differences in all-cause mortality between the diet groups. For specific causes of death, compared with regular meat eaters, low meat eaters had ∼30–45% lower mortality from pancreatic cancer, respiratory disease, and all other causes of death, fish eaters had ∼20% lower mortality from malignant cancer and ∼20% higher circulatory disease mortality, and vegetarians and vegans had ∼50% lower mortality from pancreatic cancer and cancers of the lymphatic/hematopoietic tissue. These findings were essentially unchanged on further adjustment for BMI, and generally were robust across categories of sex, smoking, and BMI for the 6 most common causes of death. When we excluded data for participants known to have changed diet group at least once during follow-up (on the basis that a change in diet group could be prompted by the onset of illness), compared with regular meat eaters, low meat eaters had ∼45% lower mortality from pancreatic cancer and ∼30% lower respiratory disease mortality, fish eaters had ∼25% lower mortality from malignant cancer, and vegetarians and vegans had ∼10% lower all-cause mortality, ∼20% lower mortality from malignant cancer, and ∼50–60% lower mortality from pancreatic cancer and cancers of the lymphatic/hematopoietic tissue. Separating the vegetarians and vegans for the 6 most common causes of death did not reveal any statistically significant differences in mortality between vegans and regular meat eaters. When we censored the data to study mortality before age 75, the results were broadly similar to those for mortality to age 90.

Our results for all-cause mortality are in line with previous studies of mortality in United Kingdom-based vegetarians and comparable nonvegetarians (5, 8, 12). In contrast, recent results from the AHS-2 study found that all-cause mortality was 12% (95% CI: 3, 20) lower in all vegetarians combined (including occasional meat eaters and persons who ate fish but not meat) than in nonvegetarians (9), and earlier studies of US Seventh-Day Adventists also found lower all-cause mortality in vegetarians (5). Orlich et al. (9) considered possible reasons for this apparent discrepancy in findings between US-based Seventh-Day Adventists and United Kingdom-based free-living subjects, suggesting that the “perceived healthfulness of vegetarian diets” may be a major motivating factor for Adventist vegetarians, whereas United Kingdom vegetarians may be motivated by other factors that are not health-related, making them less likely to adopt a “healthy” vegetarian diet (13). It may also be the case that differences in the dietary characteristics of vegetarians and nonvegetarians are greater among Adventists than among the participants in our studies. For example, nonvegetarians in the AHS-2 had 2.6 times the intake of animal protein (expressed as a percentage of energy intake) as lacto-ovo-vegetarians in the same study (14), rather higher than the ratio of 2.1 between the animal protein intake of regular meat eaters and vegetarians and vegans in the present study (Table 1). Whether such variations in diet between vegetarians and nonvegetarians in the United States and the United Kingdom contribute to the differences in all-cause mortality between vegetarians and nonvegetarians in the AHS-2 and between regular meat eaters and vegetarians in the present study is unclear.

We found no difference in IHD mortality between diet groups. This contrasts with results from the AHS-2, which showed significantly lower IHD mortality in fish eaters than in nonvegetarians (who ate meat and fish more than once per week; HR: 0.65; 95% CI: 0.43, 0.97) (9), and an earlier EPIC-Oxford study showing a 32% (95% CI: 19, 42) lower risk of incident IHD (including 1235 mostly nonfatal IHD events) in vegetarians than in nonvegetarians (15). The most likely explanation for the finding of a lower risk of incident IHD in vegetarians is that vegetarians have generally lower values of established risk factors for IHD (namely, non-HDL cholesterol, blood pressure, and BMI) than do nonvegetarians (16), but why this finding is not replicated for IHD mortality is unclear. The IHD risk factors that are affected by consuming a vegetarian diet are, proportionally, more strongly related to the risk of IHD at younger ages (17), which might explain our finding of a larger (but nonsignificant) difference in IHD mortality between regular meat eaters and vegetarians before age 75 y than for mortality up to age 90 y. Incident, nonfatal IHD may also lead to the effective medical management of established risk factors for IHD (e.g., drugs to treat non-HDL cholesterol and high blood pressure), lowering subsequent IHD mortality and partially nullifying the differences between vegetarians and meat eaters found for IHD incidence.

In our analysis of cancer incidence in British vegetarians, based on data from the same 2 cohort studies, we found an ∼10% significantly lower incidence of all malignant cancers in fish eaters and vegetarians (including vegans) than in meat eaters (11). In the present study, fish eaters had a significant ∼20% lower malignant cancer mortality than did regular meat eaters, whereas vegetarians and vegans had a nonsignificantly lower cancer mortality than regular meat eaters (by ∼10%), so our findings are consistent with the results for overall cancer incidence. In the AHS-2, there was no difference in cancer mortality between vegetarians and nonvegetarians (9). The lower mortality for pancreatic cancer and cancers of the lymphatic/hematopoietic tissue in vegetarians and vegans compared with regular meat eaters partially reflects the findings from our analysis of cancer incidence in British vegetarians (11). Although data from the EPIC study as a whole found no association between intakes of red and processed meat and pancreatic cancer risk, poultry consumption was associated with an increased risk (18), and a meta-analysis of 11 prospective studies found a positive association between pancreatic cancer incidence and processed meat consumption (19).

We also found significant heterogeneity of risks between the diet groups for circulatory disease, cerebrovascular disease, and respiratory disease mortality, although there were no obvious patterns to the HRs and the heterogeneity was nonsignificant for mortality before age 75 y. Similarly, the ∼25% lower mortality from all other causes (a catch-all category in which the most common cause of death is old age) in low meat eaters compared with regular meat eaters is hard to explain.

Strengths of the study include the large numbers of deaths (5294, including 2601 before age 75 y) in >1 million years of follow-up and the inclusion of many actual and potential confounders among the variables used for stratification and adjustment. There was also a high degree of consistency of diet group during follow-up, both among EPIC-Oxford study participants who completed the first follow-up questionnaire ∼5 y after recruitment and among the 2708 participants who contributed follow-up data from both studies.

Limitations of the study include the simple adjustment for physical activity and the uneven distribution of participants by sex (three-quarters of participants were women). The study participants are not representative of the United Kingdom population, but the mean intake of red meat in our reference group of regular meat eaters (84 g/d in men and 70 g/d in women) are similar to those of adults aged 19–64 y in the UK National Diet and Nutrition Survey (86 g/d in men and 56 g/d in women) (20).

In conclusion, our results suggest that United Kingdom–based vegetarians and comparable nonvegetarians (including people who eat fish but not meat and those who eat meat <5 times per week on average) have similar all-cause mortality. The differences by diet group found for specific causes of death merit further investigation.

## Footnotes

4 Abbreviations used: AHS-2, Adventist Health Study 2; EPIC, European Prospective Investigation into Cancer and Nutrition; FU1, EPIC-Oxford follow-up questionnaire 1; FU2, EPIC-Oxford follow-up questionnaire 2; FU3, EPIC-Oxford follow-up questionnaire 3; GP, general practice; ICD, International Classification of Diseases; IHD, ischemic heart disease; OVS, Oxford Vegetarian Study.
